# Altered Antibody Response to Epstein-Barr Virus in Patients With Rheumatoid Arthritis and Healthy Subjects Predisposed to the Disease. A Twin Study

**DOI:** 10.3389/fimmu.2021.650713

**Published:** 2021-03-11

**Authors:** Anders J. Svendsen, Marie Christine Wulff Westergaard, Anette Holck Draborg, René Holst, Kirsten O. Kyvik, Marianne A. Jakobsen, Peter Junker, Gunnar Houen

**Affiliations:** ^1^The Danish Twin Registry, Epidemiology, Institute of Public Health, University of Southern Denmark, Odense, Denmark; ^2^Department of Internal Medicine, Odense University Hospital, Svendborg, Denmark; ^3^Department of Autoimmunity and Biomarkers, Statens Serum Institut, Copenhagen, Denmark; ^4^Department of Haematology, Center for Cancer Immune Therapy, Herlev Hospital, University of Copenhagen, Copenhagen, Denmark; ^5^Department of Biostatistics, Institute of Regional Health Research, University of Southern Denmark, Odense, Denmark; ^6^Oslo Centre of Biostatistics and Epidemiology, Oslo University Hospital, University of Oslo, Oslo, Norway; ^7^Department of Clinical Research, Odense Patient data Explorative Network, Odense University Hospital, University of Southern Denmark, Odense, Denmark; ^8^Department of Clinical Immunology, Odense University Hospital, Odense, Denmark; ^9^Rheumatology Research Unit, Department of Rheumatology, Odense University Hospital, Institute of Clinical Research, University of Southern Denmark, Odense, Denmark

**Keywords:** rheumatoid arthritis, Epstein Barr virus, EBNA1 isotypes, EBNA1 titer, predisposition, twin study

## Abstract

**Objectives:** To study Epstein-Barr virus (EBV) antibody patterns in twin individuals with rheumatoid arthritis (RA) and their healthy co-twins, and to determine the heritability of antibody responses against the EBV encoded EBNA1 protein.

**Methods:** Isotypes of EBNA1 antibodies were measured in 137 RA affected- and 150 healthy twin pairs. We estimated the effect of RA and RA predisposition, anti-citrullinated antibodies (ACPA), IgM rheumatoid factor (RF), the shared epitope (SE) and the PTPN22-T allele (PTPN22) on the level of EBNA1 antibodies. We also determined the heritability of EBNA1 antibody levels.

**Results:** IgA-EBNA1 antibody levels were increased in twins from RA discordant twin pairs irrespective of RA, ACPA or IgM-RF status. The IgG-EBNA1 antibody level was elevated in healthy co-twins from RA discordant twin pairs but not in RA affected twins. The IgM-EBNA1 antibody level was elevated in both RA twins and their healthy co-twins. The effect of RA on the IgA-EBNA1 antibody level was reversed when SE was present and with no effect of PTPN22. The heritability of IgA-, IgG- and IgM-EBNA1 antibody level was 40.6, 65.5, and 54.3%, with no effect of environment shared by the twins.

**Conclusion:** EBNA1 antibody levels are distinctively different between patients with RA and healthy subjects but also between relatives of RA strongly predisposed to RA and healthy subjects. The high level of IgA EBNA1 antibodies associated with RA and a family predisposition to RA is attributable to both genetics incl. the shared epitope and environmental variation.

## Introduction

Rheumatoid arthritis is a systemic autoimmune disease which is primarily characterized by peripheral joint synovitis which if left untreated may lead to joint destruction and loss of function. The etiology is unknown, but infections have been proposed as environmental triggers in up to 20% of patients (1). Viral triggers in particular have attracted attention. Thus, polyarthritis resembling RA is common in multiple viral infections including rubella, HTLV-1, parvovirus B19 and hepatitis B and C (2). Epstein Barr virus (EBV) has long been suspected to be implicated in the pathogenesis of RA, among others because EBV has been associated with several other autoimmune diseases, e.g., multiple sclerosis and systemic lupus (3). EBV is a DNA-containing herpes virus and 80–100% of adults worldwide have serological evidence of past infection (4). The primary infection mostly occurs in childhood and thus precedes the most common age of clinical RA onset. Like RA, EBV infection is chronic with episodic flares. It has a marked tropism for B lymphocytes and exerts a wide range of immune-modulating effects including production of several pro-inflammatory cytokines (5). In addition EBV is present in synovial lining cells of chronic RA (6) and RA patients have a higher frequency and level of antibodies against specific epitopes on EBV-encoded antigens (7).

A recent meta-analysis on sero-epidemiological associations between EBV and RA did not support the hypothesis that previous infection with EBV predisposes to RA (8). However, association studies defining the occurrence of EBV antibodies as a binary serologic outcome may not reliably reflect a biologically significant impact on the RA disease pathway. Furthermore, patients with RA have a decreased T cell response to EBV gp110 (also referred to as BALF 4) which is an important EBV regulatory protein (9). Therefore, persons susceptible to RA may have an impaired control of EBV infection leading to chronic exposure to other EBV antigens and hence a sustained inflammatory response (10). Thus, reliable tests for quantification of antibodies directed against specific EBV antigens may provide a more detailed insight into the potential involvement of EBV in the RA disease process (7). EBNA1 is the only EBV protein required for maintenance of the viral genome, and when B-cells are latently infected, EBV will only express EBNA1 (11–13).

To investigate the association between, RA as well as susceptibility to RA, and EBNA1 antibody isotype levels in RA we studied cohorts of healthy twins and disease discordant twin pairs. In addition, we estimated the heritability of antibody levels and assessed a potential modifying effect of the shared epitope (SE), its QKRAA motif (14) and the PTPN22-T polymorphism (PTPN22) on the association between RA and the EBNA1 antibody profile.

## Materials and Methods

### Patients

This study was based on two twin cohorts recruited from The Danish Twin Registry (Table 1). One cohort was ascertained based on the presence of RA in at least one birth partner of a twin pair and thus comprises RA twins as well as their co-twins without RA. The diagnosis was established according to the modified ARA 87 criteria (15). The other cohort consisted of 150 healthy twin pairs born 1952 through 1982. Between 1997 and 2000 these twins underwent a structured interview and a clinical examination. Candidates were excluded if they had RA, diabetes, cardiovascular disease or any other chronic disease. All twins were of Caucasian ancestry. Detailed information on the recruitment of RA and healthy twins has previously been published (16–18).

**Table 1:** Twin pairs according to zygosity and health.

	RA complete pairs*	**RA incomplete pairs**	**Healthy complete pairs**	**Total**
Monozygotic	22	4	70	96
Dizygotic same sexed	45	21	80	146
Dizygotic opposite sexed	20	21	-	41
Total	87	46	150	283

*Complete pairs: Both twin from at twin pair participated. Incomplete pairs: only the RA twin participated*.

### Laboratory Procedures

Serum was frozen immediately at −80°C. DNA was isolated from EDTA blood samples and kept at −80 degrees. Zygosity among same-sexed twins was determined by genetic markers.

### Genotyping of HLA

HLA-DRB1 high-resolution genotyping was performed by sequencing using the HLA-DRB1 SBT Resolver kit (Olerup) followed by analysis of data using the Conexio Genomics' Assign SBT software according to the manufacturer's instructions.

The following subtypes contain the QKRAA sequence; 0401; 0409; 0413; 0416; 0421; 1419; 1421 (19).

*PTPN221 (rs2476601)* was determined with end point genotyping using TaqMan SNP Genotyping Assay kit (ThermoFisher Scientific) according to the manufacturer's instruction. PCR was run on a LightCycler 480 II (Roche) starting with an initial denaturation at 95°C for 10 min followed by 40 cycles of 95°C for 20 s; 62°C for 20 s and 72°C for 20 s.

*IgM-RF* was determined by ELISA using purified IgG as antigen and peroxidase-conjugated F(ab)2 rabbit immunoglobulin (DAKO, Copenhagen, Denmark) against human IgM as detecting antibody. Detection limit: 1.4 IU/ml, cut off value: 17 IU/ml, clinical sensitivity for RA: 70%, clinical specificity for healthy donors: 95%.

*Anti-cyclic citrullinated peptide (CCP) IgG antibodies (ACPA)* were determined by ELISA (Euro-Diagnostica, Malmö, Sweden) as described by the manufacturer. Clinical sensitivity for RA: 77.4%, clinical specificity for healthy donors: 98.8%. Detection limit 1.6 U/ml, cut off value 25 U/ml.

*EBNA1 antibody ELISA was determined as previously described* (20).

Briefly, TTN buffer (0.025 M Tris, 0.5% tween 20, 0.15 M NaCl, pH 7.4) was used for blocking, dilutions and washings. Recombinant EBNA1 (1.0 μg/ml, *E.coli*-derived, EBV-271, Prospec Protein Specialist, Ness-Ziona, Israel) diluted in carbonate buffer (50 mM sodium carbonate, by pH 9.6) was coated on to NUNC Polysorp microtiter plates (Thermo Fisher Scientific, Roskilde, Denmark) overnight at 4°C for EBNA1 IgG and IgM detection and at room temperature (RT) for IgA detection. After coating, plates were washed 3 × 1 min followed by blocking for 30 min. The samples were subsequently incubated for 1 h at RT in duplicate in both coated and non-coated wells diluted 1:1,000/1:150/1:200 for detection of EBNA1 IgG/IgA/IgM. Following another three washings, the plates were incubated for 1 h at RT with alkaline phosphatase (AP)-conjugated goat antihuman IgG, IgA or IgM (diluted 1:5,000, 1:1,000 or 1:5,000, respectively, Sigma-Aldrich, St. Louis, Missouri, USA). After three more washings, antibodies were quantified using ρ-nitrophenylphosphate (ρ-NPP) (1 mg/ml, Sigma-Aldrich, St. Louis, Missouri, USA) dissolved in AP substrate buffer (1 M diethanolamine, 0.5 mM MgCl_2_, pH 9.8). After incubations for 30 min for measuring EBNA1 directed IgG antibodies and after 60 min for measuring IgA and IgM antibodies, the absorbances were read on a microplate reader at 405 nm, with background subtraction at 650 nm.

For each sample, the absorbance values of non-coated wells were subtracted from the coated wells after averaging the duplicates. In each plate a standard curve was included derived by two-fold serial dilutions of high antibody titer plasma pools. Furthermore, a low positive and a high positive control as well as a negative control was included in each plate. All net absorbance values were normalized relative to this standard creating arbitrary antibody titer concentrations (U/ml) for included samples. Samples with a calculated titer > 100 U/ml were rerun and normalized relative to the standard.

Inter-assay coefficients of variation were 17.9, 7.3, and 14.0% regarding EBNA1-directed IgG, IgA, and IgM, respectively, calculated from the low positive control. Intra-assay coefficients of variation were 9.2, 4.9, and 5.7% regarding EBNA1-directed IgG, IgA, and IgM, respectively, calculated from 16 values derived from the same sample in each assay (a sample known to have an antibody titer in the linear part of the standard curve).

### Statistical Analysis

Regression models for the three isotypes were adjusted for age, gender and the potential effect of RF, ACPA, and RA. Furthermore, we tested the potential effect of belonging to a twin pair, with at least one twin having RA. We included this effect because we wanted to study if the healthy co-twins from RA discordant twin pairs shared an underlying unobserved effect on the level of EBNA1 antibodies. This was done as healthy co-twins have a relative risk of developing RA of 25 compared to the background population reflecting a high degree of liability to RA in these co-twins (16, 17).

The SE, and its QKRAA subtype, and the PTPN22 C1858T-allele (PTPN22) was measured in pairs with at least one twin being diagnosed with RA. This was done to assess whether the effect of RA on EBNA1 anti-body level was modified by the presence of SE or PTPN22, which are the two major genetic risk factors in RA.

The inflammatory condition in RA may lead to high concentrations of immunoglobulins, including those directed against EBV, as well as rheumatoid factor (RF). We have therefore adjusted for the presence of RF in the analysis.

Twin data can be used to study the heritability of traits by use of the so called ACDE model, where differences in the covariance structures between DZ and MZ twin pairs contrast the genetic and environmental contributions (21, 22). The model allows for assessment of the proportion of total variance that can be attributed to genetics and environment shared by the twins and can readily be fitted by standard statistical software packages (23, 24). In the present study we used the xtmixed command software in Stata, Ver. 14.2.

The ACDE model for analysis of twin data, is a regression-type model, with a covariance structure that reflects the assumed intra-pair correlations induced by additive (A) and dominant (D) genetic effects and common (C) and individual (E) environmental effects. The E component is merely a term for the measurement error known from conventional regression models. Most data do not allow for estimating both genetic components. A pragmatic approach is therefor to fit both an ACE model and a DCE model and choose the one, which provides the best fit. In addition to estimating the effect of some covariates, the ACDE model also allows for estimating the heritability of a trait defined as the proportion of the total random variation that can be attributed to genetic variation.

Stata Ver. 14.2 for windows was used for the statistical analyses and a *p*-level < 0.05 was chosen as statistically significant.

#### Ethics

The study was approved by all the regional scientific ethics committees in Denmark (Projekt ID: S-20070088) and the Danish data protection board (J.nr. 2007-41-0747).

## Results

### Ascertainment and Characteristics of Twins

A total of 283 twin pairs participated in this study, 150 healthy pairs and 133 RA-affected twin pairs (Table 1).

Among the RA pairs 87 were complete (both twins participated) and 46 were incomplete in which only the RA-affected twin participated. Two monozygotic (MZ) and 2 dizygotic same sexed (DZss) pairs, all females, were concordant for RA. Thus, 137 RA affected twins and 83 of their healthy co-twins in addition to 300 twins from healthy pairs participated.

The mean age of RA onset was 44 years and the mean age at blood collection was 63.5, 61.7, and 35.2 in RA twins, healthy RA co-twins and healthy twins, respectively (Table 2).

**Table 2:** Characteristics of twin individuals.

	**RA twins**	**Healthy RA co-twins**	**Healthy twins**
Mean age at onset (years), range, SD	44.0 (15–72, SD 13.4)		
Mean age at sample (years), range, SD	63.5 (30–78, SD 10.9)	61.7 (28–78, SD 11.8)	35.2 (18–45, SD 7.2)
Male sex (%)	25.5	43.2	46.0
IgM-RF positive (%)	67.2	7.2	1.2
Anti- CCP positive (%)	76.0	18.9	2.6
Shared Epitope positive (%)	75.2	64.2	-
QKRAA positive (%)	51.1	32.6	-
PTPN-22 T-allele positive (%)	30.7	23.4	-

In RA twins 74.5% were females and the sex distribution and the occurrence of erosive or nodular disease were similar across zygosities (16). The two zygosity groups were also comparable regarding smoking history, presence of autoantibodies, SE and PTPN22. The proportion of rheumatoid factor, ACPA, SE, and PTPN22 positive twins was 67.2, 76.0, 75.2, and 30.7% among the RA twins and 7.2, 18.9, 64.2, and 23.4% among their healthy co-twins. In the healthy twin pairs the prevalence of RF (2.1%) and ACPA (1.4%) was comparable to the prevalence in the background population of singletons and the population prevalence of SE and PTPN22 is 52% (25) and 21% (26).

### EBNA1 Titer According to Antibody Isotype

**Figure 1:**
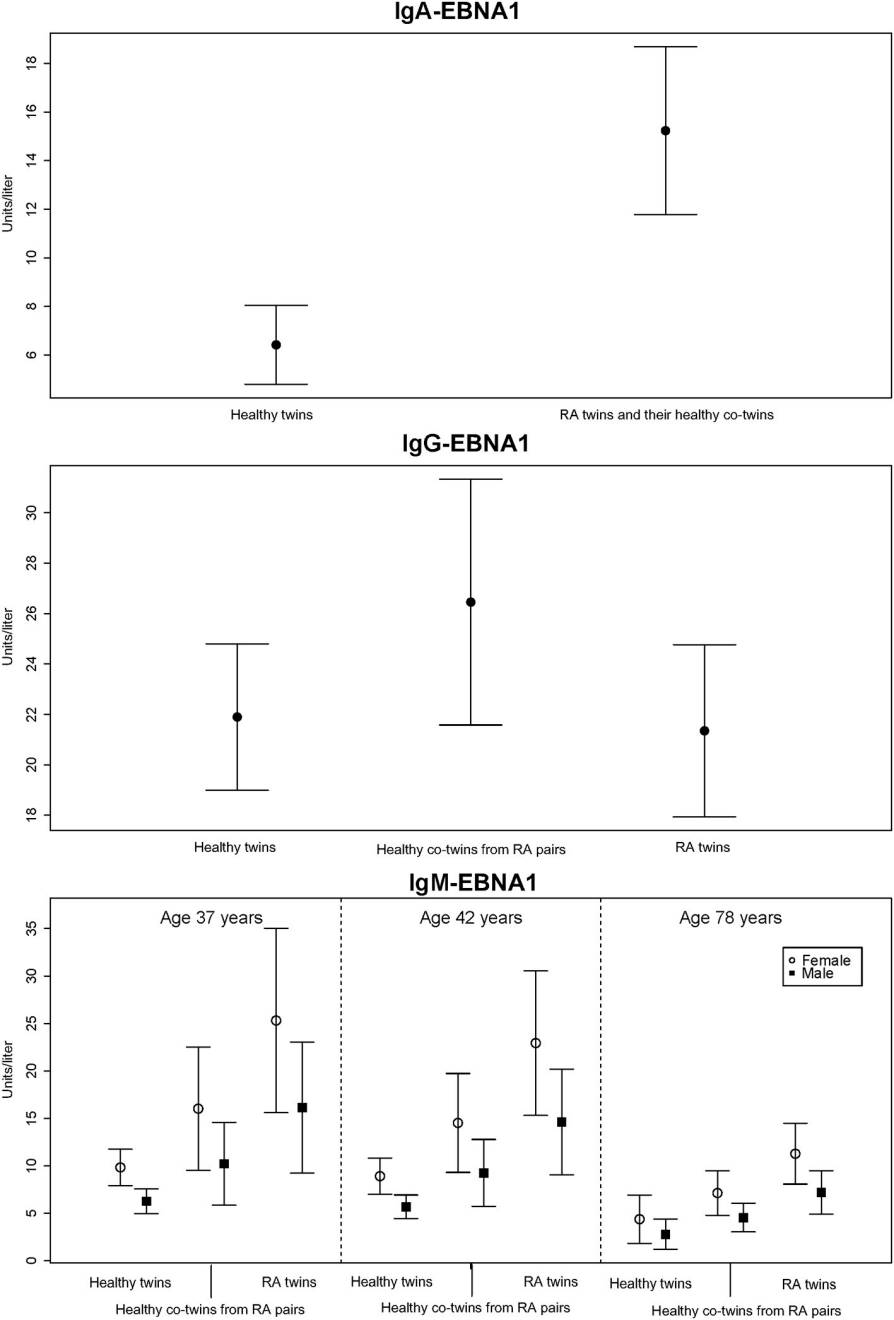
The mean level of EBNA-1 antibodies (units/liter) according to immunoglobulin isotype class with 95% error bars.

RA-affected twin pairs had significantly increased IgA-EBNA1 titers whereas there was no effect by RA itself or any of the covariates RF, ACPA, age or sex. Since there was no difference in IgA-EBNA1 antibody levels between twins with RA and their healthy co-twin we pooled the results (Figure 1 and Table 3). The heritability estimate of the IgA-EBNA1 titer was 40.6% and the effect of the unique environment was 59.4% with no effect of shared environment.

**Table 3:** Co-variates predicting EBNA1 antibody levels.

**EBNA1 isotype**	**Co-variate**	**Regression coefficient**	***P*-value**	**95% CI interval**
IgA-EBNA1	RA twin pairs	0.7122	<0.000	0.4198 to 1.0047
IgG-EBNA1	RA	−0.4446	<0.002	−0.7273 to −0.1620
	RA twin pairs	0.6263941	<0.015	0.1211 to 1.1317
IgM-EBNA1	RA twin pairs	0.4762	<0.024	0.0617 to 0.8927
	Male Sex	−0.3799	<0.001	−0.6098 to −0.1500
	Age	−0.0145	<0.022	−0.0269 to −0.0021
IgM-EBNA1	RA	0.3572	<0.006	0.1040 to 0.6105
	Male Sex	−0.3556	<0.003	−0.5874 to −0.1238
	Age	−0.0082	<0.044	−0.0162 to −0.0002

The IgG-EBNA1 titer was significantly higher in healthy co-twins from RA affected twin pairs vs. their RA counterparts and healthy controls. There was no effect of RF, ACPA, age or sex (Figure 1 and Table 3). There was a genetic effect on the IgG-EBNA1 concentration with a heritability estimate of 65.5%, an effect of the unique environment of 34.5% and no effect of shared environment.

Both RA-affected twins and their healthy co-twins from RA-affected twin pairs had significantly elevated IgM-EBNA1 titer compared to healthy twin pairs. Belonging to an RA-affected twin pair fitted marginally, but insignificantly better than a model including RA-status. Male sex and increasing age were associated with a lower IgM-EBNA1 titer (Figure 1 and Table 3). The heritability was 54.3% and unique environment explained the remaining 45.7%.

### Effect of SE and the PTPN22 on the IgA-EBNA1 Levels

We found significant effect modifications of SE and the QKRAA subtype on the association between RA and the level of IgA-EBNA1 antibodies but not the other isotypes. The regression coefficient of RA was −0.8844 (95% CI: −1–4909 to −0.1978, *p* < 0.01) in SE negative RA and 0.3175 (95% CI: −0.1227 to 0.7579, *p* < 0.16) in SE positive RA and −0.3459 (95% CI: −0.8219 to 0.1302, *p* < 0.15) in QKRAA negative RA and 0.5126 (95% CI: −0.0844 to 1.1096, *p* < 0.09) in QKRAA positive RA. Inclusion of the interaction terms resulted in significantly better model fits. Thus, we observed a crossover interaction with reversal of the RA effect when SE and QKRAA was present. That is, the regression coefficient of RA on IgA-EBNA1 antibodies was negative in SE negative RA and QKRAA negative RA and positive in SE positive and QKRAA positive RA. Hence, the effect of RA on IgA-EBNA1 level goes in opposite directions in SE (QKRAA) positive RA compared to SE (QKRAA) negative RA. In other words, the presence or absence of the shared epitope determines whether the effect of both RA *per se* and RA disposition, increases or decreases the level of IgA-EBNA1 antibodies.

We observed a significant negative effect of PTPN22 on the IgA-EBNAR1 level but unlike the SE, PTPN22 did not interact with RA.

## Discussion

This study shows that the serum levels of EBNA1 isotype antibodies differ in patients with RA and in their unaffected co-twins as compared with healthy subjects. Of particular note, IgA-EBNA1 antibodies are significantly increased in both twin individuals from RA discordant pairs. The altered IgA-EBNA1 antibody response is modified by the presence of SE. Genetics account for ~50% of the variation in EBNA1 antibody level regardless of anti-body isotype.

The IgA-EBNA1 antibody levels were equally increased in twin individuals with RA vs. their clinically healthy co-twins with no effect of age, sex, IgM-RF or ACPA on the antibody levels, implying that RA *per se* is not of primary significance for this aberration. Our findings accord well with our previous report that the relative risk of developing RA when having an affected co-twin compared to the background population risk is highly increased amounting to 24.6–35.4 in MZ twins and 17.3–31.6 in DZss twins (16). Since our study was based on prevalent RA cases previously or currently treated with immunosuppressive agents, it could be argued that perturbations of the immune system associated with RA itself may have been modified by drug actions. However, so far no evidence has been provided that there is an association between EBNA1 antibody levels and immunosuppressive treatment, including methotrexate (MTX) (20). Additionally, since only RA twins but not healthy co-twins had been treated, the difference in IgA-EBNA1 antibody level between healthy, but RA-predisposed co-twins, and the population of healthy twins cannot be ascribed to an effect of treatment.

A recent study reported that IgA plasmablast numbers are elevated in subjects at risk of acquiring RA compared to healthy controls and early RA (27). It remains to be determined if the high IgA-EBNA1 antibody level is part of a non-specific mucosal immune response in RA or if EBV is specifically involved.

IgA antibodies play a crucial role in the immune function of mucous membranes and detection of IgA antibodies is often considered as a measure of epithelial infection load. Hence, the finding of high IgA-EBNA1 antibodies in RA affected twin pairs could be a consequence of a higher viral load, perhaps in epithelial cells in particular. It would therefore be relevant to measure the viral load in e.g., saliva but also in synovial biopsies and/or synovial fluid from cases and controls. The observation of equally high IgA titers in healthy co-twins and in RA twins indicates that this could be a part of the immunological pathway leading to RA or be a consequence of RA induced perturbation of immunological pathways. We found that genetic effects accounted for 40% of IgA-EBNA1 levels and that unique environmental effects accounted for the remaining 60%, as we did not detect any effect of shared environment between the twins.

Family predisposition to RA was also associated with increased IgG EBNA1 antibody levels which unlike IgA-EBNA1 antibodies, only occurred in the non-RA co-twins (Figure 1). Although current or previous DMARD therapy would appear to be a plausible explanation for this discrepancy, previous studies have reported that EBV load and the EBNA1-IgG antibody response are left unaffected by treatment with MTX or TNF-inhibitors (20, 28, 29).

Two previous studies reported higher levels of IgG-EBNA1 antibodies in patients with RA (30, 31) whereas three other studies did not (32–34), while a large Swedish study reported a lower level, particularly in ACPA-positive RA, and with no impact of RF (35). A study by Westergaard et al. reported elevated EBNA1 antibodies of all three isotypes in RA but their results were based on a stratified analysis unadjusted for age and sex (20). Compared to the study by Westergaard et al. we have undertaken a more thorough analysis adjusting for both sex, age, RF, ACPA, and SE simultaneously in our multiple regression analysis. Westergaard et al. only found an association with RF and ACPA with IgM-EBNA1 and IgA-EBNA1 antibody isotypes and not IgG-antibodies. In addition, the Swedish study reported no effect of RF but a small effect of ACPA but only concerning IgG-EBNA1 antibodies (35).

In two prospective studies on preclinical RA, blood samples collected 5.6 and 9.3 years before clinical disease onset titers of IgG-EBNA1 antibodies were not increased (29, 36) indicating that increased EBV antibody levels are more likely a consequence of an immune system dysregulation later on in the preclinical phase of the disease course. Our RA affected twin pairs had on average been disease-discordant for 19 years and we do not know the true induction time (the time from the beginning of the disease process and appearance of clinical disease) in RA and if aberrant immune responses involved in the RA pathogenesis reduces the IgG-EBNA1 antibody production it may more likely occur in individuals with more imminent RA. A previous study reported that seropositivity for IgG-EBNA1 antibodies did not differ between RA and controls up to 23 years before diagnosis (29). These studies did not investigate other EBV antibody isotypes, whereas one of the studies reported that certain cytokines were upregulated within the 5 year pre-diagnostic time window and that the presence of IgG-ACPA and IgM- and IgA-rheumatoid factors may precede RA by at least two decades indicating a long induction period (29).

We found that RA and RA predisposition were associated with almost equally increased IgM-EBNA1 antibody levels (Figure 1). Both male sex and increasing age was associated with declining levels of IgM EBNA1 antibodies. It is uncertain if these age related trends affect the antibody avidity and functional capacity (37).

The heritability of EBNA1-IgG and -IgM antibodies was estimated at 65 and 54% with no effect of shared environment. Thus, the genetic variation contributing to RA predisposition seems to account for most of the variation in antibody level across EBNA1-antibody isotypes.

The SE is strongly associated with ACPA-positive RA and subsets of the SE contain the QKRAA sequence which is homologous with the EBV protein gp110 (14). Synovial EBV DNA loads are highest in patients with RA with at least one copy of the SE and are associated with low frequencies of T cells specific for the EBV gp110 glycoprotein which is crucial to control of EBV infection (38). This may indicate a partial T-cell tolerance to EBV in SE positive subjects. We found that the presence of SE and QKRAA reversed the effect of RA on the IgA-EBNA1 antibody response. Thus, the higher IgA-EBNA1 antibody response in SE positive RA may reflect a compensatory mechanism to T-cell tolerance. This finding may support the molecular mimicry hypothesis (39). There was no interaction between SE and the levels of IgM- and IgG-EBNA1 isotypes. The latter finding accords with a previous study on IgG antibodies against EBNA1 in preclinical RA (36).

Some limitations should be considered. This is a cross-sectional study investigating twins with established RA. Hence, it cannot be definitely determined if the variation of the EBNA1 antibody level is a cause or a consequence of RA. However, the equally elevated levels of IgA-EBNA1 antibodies in RA twins and their healthy co-twins support the basic thesis that EBV infection is involved in the RA pathogenesis or that aberrant disease processes leading to RA may have modified the immune response to EBV.

If we had had the opportunity to measure the isotype of VCA antibodies we might have obtained indication if lytic activity was going on. Nor did we have any synovial biopsies, synovial fluid or saliva samples and we were therefore unable to investigate if the antibody level is related to viral load.

Our finding of high IgA-titers in the blood incites further exploration e.g., direct sequencing fluorescence *in-situ* hybridization of e.g., synovial biopsies, PCR analysis of blood samples for EBV-derived DNA and RNA, and perhaps even more relevant analysis of simultaneously obtained saliva samples, as IgA antibodies was the most prominent finding in our study (40). Studies on synovial fluid may also be relevant as it is well-documented that synthesis of IgA isotypes may exist in synovial cells (41).

The healthy twin population was younger than the RA twin population. Yet, the IgG-EBNA1 level has been reported to remain constant in adulthood in healthy individuals (42) as well as in patients with multiple sclerosis (43). Besides, we did not observe an effect of age on the IgA- and IgG-EBNA1 antibody level and only a slight age-related decrease of the IgM-EBNA1 level. Furthermore, the level of EBNA1 antibodies of all three isotypes has previously been observed to be significantly higher in singletons with RA compared to healthy controls (20). Although the prevalence of autoantibodies, including IgM-RF, increases with age independent of sex (44), comparable levels of IgM-RF have been observed across age categories in the general population (45). Thus, it is unlikely that the mean age difference between the RA affected twin pairs and the healthy twin pairs constitutes a significant source of concern.

A major strength of this study was the twin design which enabled us to distinguish between the effect of established RA and predisposition to the disease and to estimate the heritability of the variation of EBNA1 antibody levels.

In conclusion, the humoral EBNA1 immune response to EBV is altered both in individuals with RA and in their clinically healthy co-twins. The patterns of circulating IgA, IgG, and IgM antibodies differ distinctively between a healthy reference population, individuals predisposed to RA and RA patients. Presence of SE modifies the IgA-EBNA1 antibody response pattern. Genetics account for ~50% of EBNA1 isotype antibody variation. The level of IgM-EBNA1 has already been suggested as a potential marker to distinguish RA from systemic lupus (46) and prospective studies are needed to elucidate if an increased IgA-EBNA1 antibody level in healthy relatives to RA patients may serve as an early serological risk marker for future RA development.

## Data Availability Statement

The raw data supporting the conclusions of this article will be made available by the authors, without undue reservation.

## Ethics Statement

The studies involving human participants were reviewed and approved by all the regional scientific ethics committees in Denmark (Projekt ID: S- 20070088) and the Danish Data Protection board (J. nr. 2007-41-0747). We obtained informed written consent from all participants in the study. The patients/participants provided their written informed consent to participate in this study.

## Author Contributions

AS conceived the study. AS, RH, PJ, and GH analysis and interpretation of the data and prepared the manuscript. GH, MW, AD, KK, and MJ contributed reagents/materials/analysis tools. All authors contributed to the article and approved the submitted version.

## Conflict of Interest

The authors declare that the research was conducted in the absence of any commercial or financial relationships that could be construed as a potential conflict of interest.
